# Metastasis of colorectal cancer to the uterine body and fallopian tube: case report and literature review

**DOI:** 10.1093/jscr/rjae400

**Published:** 2024-06-10

**Authors:** Sergey K Efetov, Yu Cao, Jinqi Zou, Anton Y Dorogov, Nina B Paramonova, Larisa V Tsoy, Inna V Droshneva, Anastasia S Fatyanova

**Affiliations:** Department of Faculty Surgery No.2, I.M. Sechenov First Moscow State Medical University (Sechenov University), Moscow, 119435, Russia; Department of Faculty Surgery No.2, I.M. Sechenov First Moscow State Medical University (Sechenov University), Moscow, 119435, Russia; Department of Faculty Surgery No.2, I.M. Sechenov First Moscow State Medical University (Sechenov University), Moscow, 119435, Russia; Department of Faculty Surgery No.2, I.M. Sechenov First Moscow State Medical University (Sechenov University), Moscow, 119435, Russia; Insitute for Clinical Morphology and Digital Pathology, I.M. Sechenov First Moscow State Medical University (Sechenov University), Moscow, 119435, Russia; Insitute for Clinical Morphology and Digital Pathology, I.M. Sechenov First Moscow State Medical University (Sechenov University), Moscow, 119435, Russia; P.A. Herzen Moscow Oncology Research Institute, Branch, Nation Medical Radiology Research Center, Ministry of Health of Russia, Moscow, 119435, Russia; Department of Oncology, Radiotherapy and Reconstructive Surgery, I.M. Sechenov First Moscow State Medical University (Sechenov University), Moscow, 119435, Russia

**Keywords:** colorectal cancer, metastatic tumors, fallopian tube, case report

## Abstract

Colorectal cancer typically metastasizes to the peritoneum, liver, and lungs. However, metastases to the fallopian tube and uterus are uncommon. This case report delves into this rare occurrence of metastasis and discusses its characteristics, diagnostic methods, and treatments based on an extensive literature review. We present the case of a 61-year-old female patient who underwent her initial hospitalization for da Vinci robotic surgery to address colorectal cancer, stage pT3N0M0. However, during routine postoperative follow-up 6 months later, a localized rectal recurrence was detected. The patient commenced chemoradiotherapy with full response. Subsequently, the patient was readmitted due to pelvic pain again, and a magnetic resonance imaging scan revealed an abnormal mass in the patient’s left fallopian tube and uterine corpus, infiltrating the myometrium. Consequently, total hysterectomy with bilateral adnexectomy was performed, along with omentectomy, which confirmed metastatic involvement from rectal cancer upon postoperative pathological examination. This case may inform further diagnosis and treatment of colorectal cancer metastasis to the fallopian tube.

## Introduction

Colorectal cancer (CRC) exhibits a propensity for metastasis, predominantly to the liver [1]. The primary route of dissemination is hematogenous, typically via the portal circulation. Additionally, CRC commonly disseminates to the lungs, peritoneum, and lymph nodes [2, 3]. Metastatic involvement serves as an adverse prognostic factor, and surgical intervention has demonstrated efficacy in enhancing the 5-year survival rate, achieving a rate of 71% [4]. Metastatic fallopian tube cancer (MFTC) is rare, with recorded cases suggesting that ~30% originate from CRC [5].

## Case report

**Figure 1:**
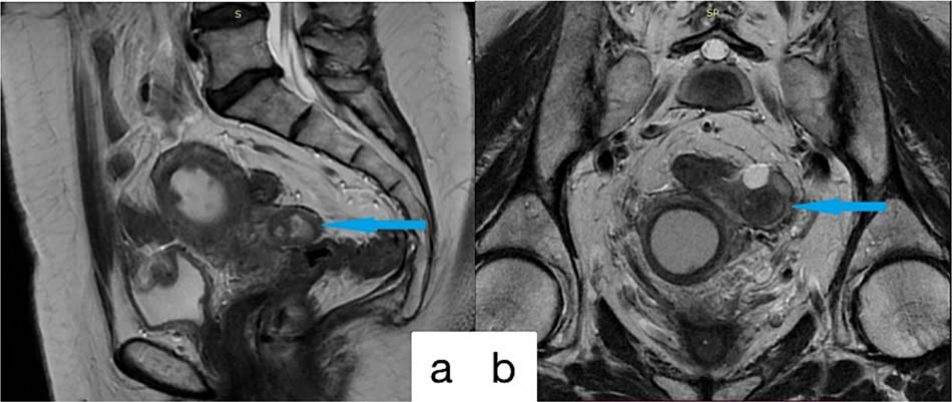
Preoperative MRI imagines; (a) sagittal; (b) coronal; alterations in the left fallopian tube, enlargement of the uterine cavity and cervical canal, and soft tissue hyperplasia within the uterine cavity.

**Figure 2:**
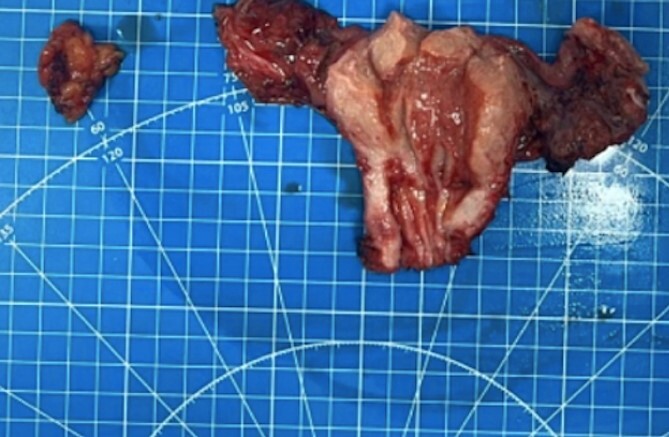
The obturator lymph node, uterus, and fallopian tube from the patient were received, revealing a significant tumor presence.

A 61-year-old female patient was admitted to the hospital presenting with lower abdominal discomfort and purulent discharge from the genital tract. The patient was diagnosed CRC 3 years ago, staged as pT3N0M0. She underwent robotic low anterior resection with D3 lymph node dissection as primary treatment, followed by surveillance without adjuvant chemoradiotherapy. At the 6-month postoperative follow-up, local recurrence at the anastomotic site was detected, prompting initiation of radiotherapy with a total load of 50 Gray and chemotherapy with capecitabine 1000 mg/m^2^ per day for 2 months anв resulted in full response. Subsequent Magnetic Resonance Imaging (MRI) evaluation posttreatment revealed complete clinical remission. During 17 months follow-up after surgery, MRI revealed no evidence of disease progression in the colon, alterations in the left fallopian tube, enlargement of the uterine cavity and cervical canal, and soft tissue hyperplasia within the uterine cavity (Fig. 1). The result of laboratory tests revealed no breast cancer gene 1 (BRCA1) or BRCA2 gene mutations, Carbohydrate antigen 19–9 (CA 19–9) levels at 12.7 U/ml, Carcinoembryonic antigen at 1.3 ng/ml, and cancer antigen 125 (CA-125) at 63.2 U/ml. Uterine cavity biopsy identified G2 adenocarcinoma growth in the uterine corpus. During a subsequent surgical procedure, the presence of a tumor in the left fallopian tube was identified, leading to an enlarged resection of the uterus and adnexa (Fig. 2). Histopathological examination indicated tumor invasion of the left fallopian tube, along with infiltration into the superficial myometrial layers, not exceeding 1 mm, wall, particularly in the endo-tubal region.

**Figure 3:**
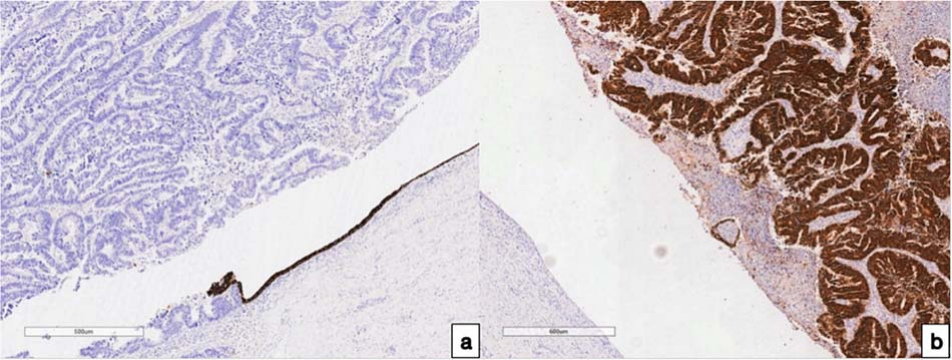
Immunohistochemical examination of the fallopian tube tumor; (a) with antibodies to CK 7, 100× magnification; (b) with antibodies to CDX 2, 100× magnification, and IHC analysis revealed positive CK 7 expressions in the preserved endometrium, while expression was notably absent in the tumor cells; conversely, positive CDX 2 expression was observed in the tumor cells, with an absence of expression noted in the endo-salpinx.

In order to determine the tumor origin, immunohistochemical (IHC) analyses (Fig. 3) and hematoxylin and eosin staining (HE) were conducted. The HE revealed adenocarcinoma growth in the fallopian tube wall. Subsequent IHC examination on demonstrated diffuse expression of Caudal type homeobox 2 (CDX 2) and Keratin 20 (CK 20) in tumor cells within the fallopian tube and uterine corpus. Conversely, Keratin 7 (CK 7) exhibited negative staining in tumor cells within the fallopian tube. However, CK 7 displayed positive staining in preserved endo-salpinx and endometrium cells. Both HE and IHC (CK 20+/CK 7-, CDX 2+ in tumor cells) observations collectively support the conclusion that the tumor metastasized to the fallopian tube from colorectal cancer.

The standard CAPOX (Capecitabine-Oxaliplatin) chemotherapy regimen in the adjuvant setting was initiated during the fourth week following the operation, with the intention of completing eight cycles. However, at the time of reporting this case, only five cycles had been successfully administered, with no indications of tumor recurrence observed during the 6-month postoperative follow-up examination. Adjuvant chemotherapy is ongoing.

## Discussion and literature review

Primary fallopian tube cancer (FTC) is a rare malignancy, with an incidence of less than 5 cases per million women per year [6]. MFTC are highly tended to be ignored in clinic practice, as its mechanism are still not totally clearly yet. However, some research indicates that lymphogenic, hematogenous, or transcavitary might serve as potential metastatic pathways [7]. Metastasis of non-gynecological malignancies to the fallopian tubes is also infrequent, with only one documented case of CRC metastasis found in the literature [8]. In a case series encompassing 20 cases of fallopian tube metastasis from non-gynecological malignancies, only 2 cases reported were secondary to CRC, while the remaining cases were suggested from other origins [9]. While the predominant metastatic sites of CRC are livers and peritoneum, there is research indicates that CRC (35%) and breast cancer (15%) are the two primary tumors that metastasize to the fallopian tube. However, metastases to the fallopian tube are still rare and are therefore often overlooked in clinical diagnosis. In addition, visible nodules are present in only 35% of cases in the CRC metastasis to the fallopian tube, significantly compromising the clinical diagnostic efficiency [10].

FTC typically presents asymptomatically, although clinical manifestations may include abdominal pain, vaginal bleeding, and abnormal discharge [11]. In this case, the patient was readmitted to the hospital due to pelvic pain and revealed a urinary fistula-a finding unusual in patients with MFTC, differing from the typical presentation of FTC. MRI is still the current gold standard for the diagnostic evaluation of indeterminate adnexal masses in the context of pelvic gynecological oncology [12, 13]. Although left ovarian lesions were detected on MRI, left FTC was always confirmed only during subsequent surgery. However, in cases where there is a large uterine mass, resulting in distortion of the normal anatomical features, the fallopian tubes may not be adequately visualized on MRI, leading to potential underdiagnosis of fallopian tube malignancies. Following imaging diagnosis, biomarkers such as CA125 are often utilized to aid in confirming the diagnosis of FTC. Nonetheless, CA125 levels are not specific, and it may be elevated in various abdominal malignancies, thereby diminishing its specificity and necessitating further diagnostic modalities for an accurate identification of fallopian tube malignancies [14].

We followed the guidelines of National Comprehensive Cancer Network plan to administer CAPOX treatment after surgery [15]. During the 6-month follow-up, no evidence of recurrence was observed. The favorable treatment outcome suggests that our treatment approach may serve as a valuable template for managing similar rare clinical scenarios in the future. Although the patient underwent treatment immediately, the early detection of secondary FTC through more effective diagnostic methods could potentially prevent the need for a second surgery and significantly improve quality of life.

In future clinical practice, the integration of artificial intelligence and personalized therapy may prove to be a feasible approach. Utilizing MRI-based deep learning (DL) could assist in accurately identifying the site of CRC metastasis [16]. Through DL technology, more comprehensive treatment plans could be achieved and thereby providing better guidance to surgeons, especially in cases of ambitious radiological findings prior operation [17]. Additionally, the personalized therapy can utilize genetic testing to identify pharmaco-resistant genes, enabling targeted therapeutic interventions [18]. Moreover, with the advancement of DL technique，the combination of DL and personalized therapy could offer promising outcomes, significantly facilitating the optimal treatment strategy selection and enhancing the overall survival of the patients. Nevertheless, crafting machine learning algorithms and constructing artificial intelligence models for rare clinical observations pose significant challenges. This highlights the importance of meticulously documenting each rare case.

## Conclusion

Metastasis of CRC to the fallopian tube is frequently disregarded by attending physicians owing to its rarity and subtle clinical manifestations. It is anticipated that the clinical insights and therapeutic experiences derived from this case will benefit future patients in clinical practice.

## Data Availability

The data generated in the present study may be requested from the corresponding author.
